# Understanding the relationship between smartphone distraction, social withdrawal, digital stress, and depression among college students: A cross-sectional study in Wuhan, China

**DOI:** 10.1016/j.heliyon.2024.e35465

**Published:** 2024-07-30

**Authors:** Yufei Qiu, Xueyang Zhao, Jiali Liu, Zhaoyang Li, Man Wu, Lixin Qiu, Zhenfang Xiong, Xiaopan Wang, Fen Yang

**Affiliations:** a School of Nursing, Hubei University of Chinese Medicine, Wuhan, 430065, China; b Ningbo Municipal Hospital of Traditional Chinese Medicine (TCM), Affiliated Hospital of Zhejiang Chinese Medical University, Ningbo, 315010, China; c School of Psychology, Center for Studies of Psychological Application, Guangdong Key Laboratory of Mental Health and Cognitive Science, Cognition and Education Sciences (South China Normal University), Ministry of Education, South China Normal University, Guangzhou, 510631, China; d School of Nursing and Health Management, Wuhan Donghu University, Wuhan, 430212, China; e Department of Nursing, Hubei Provincial Integrated Traditional Chinese and Western Medicine Hospital, Wuhan, 430015, China; f Hubei Shizhen Laboratory, Wuhan, 430065, China

**Keywords:** Smartphone distraction, Depression, Social withdrawal, Digital stress, Chain mediating model

## Abstract

**Background:**

Smartphone distraction considerably affects the depression level of college students. These two variables are highly associated with social withdrawal and digital distress. However, the underlying mechanisms of how social withdrawal and digital stress were involved in the relationship between smartphone distraction and depression remain unclear.

**Methods:**

A cross-sectional survey was conducted in seven colleges of Wuhan, Hubei Province, from September to November 2021. Participants were selected using convenience sampling. Smartphone distraction, social withdrawal, digital stress, and depression level were assessed using the Smartphone Distraction Scale (SDS), 25-item Hikikomori Questionnaire (HQ-25), Multidimensional Digital Stress Scale (DSS), and the Patient Health Questionnaire-9 (PHQ-9), respectively. All scales demonstrated good reliability in this study, the reliability of each scale was 0.920, 0.884, 0.959, and 0.942.

**Results:**

The final analysis included 1184 students (692 males and 492 females), aged between 17 and 37 years. Participants were from various academic disciplines, including medical and non-medical. The findings revealed that smartphone distraction had a significant direct effect on depression (c = 0.073, 95 % CI: 0.037 to 0.108, *p* < 0.001) and three significant indirect mediation effects: (1) social withdrawal (B = 0.083, 95 % CI: 0.066 to 0.101, *p* < 0.001), accounting for 27.76 % of the total effect; (2) digital stress (B = 0.109, 95 % CI: 0.088 to 0.132, *p* < 0.001), accounting for 36.45 % of the total effect; and (3) the chain mediating roles of social withdrawal and digital stress (B = 0.034, 95 % CI: 0.026 to 0.043, *p* < 0.001), accounting for 11.37 % of the total effect. The total mediating effect was 75.59 %.

**Limitations:**

This study is based on cross-sectional data, which limits the causality inference.

**Conclusions:**

These findings suggest that educational institutions should identify college students with excessive smartphone use early and provide timely interventions to minimize negative outcomes. It is also significant to reduce the risk of social withdrawal and digital stress to maintain the physical and mental health development of college students.

## 1 Introduction

Depression has become a major concern for the mental health of college students in China. A comprehensive meta-analysis encompassing 113 studies showed that the prevalence of depression in this demographic has reached 28.40 % [1]. Factors contributing to this include academic pressure [2,3], interpersonal relationship challenges [3], health concerns [4], and so on. Additionally, problematic smartphone usage such as Smartphone Distraction (SD) and addiction are increasingly recognized as contributing factors [5]. The impact of depression extends beyond academic disruptions; in severe cases, it can escalate to clinical depression, leading to self-harm or even suicidal behaviors [6]. It is crucial to understand the underlying factors and mechanisms contributing to depression among college students to develop timely and effective interventions.

### 1.1 SD and depression

SD has been identified as a risk factor for depression [5]. SD, which involves attention frequently being diverted from the present moment due to digital device use, plays an important role in the emergence of depression among college students [[7], [8], [9]]. Demerouti (2001) proposed the Job Demands-Resources (JD-R) model [10], which offers a valuable theoretical framework for understanding the relationship between SD and depression. According to the JD-R model, stress arises from an imbalance of demands over available resources. In the context of SD, this imbalance occurs when cognitive resources are overstretched by digital demands [11,12], potentially impairing a student's ability to meet academic and social expectations. This resource deficiency is a critical pathway through which SD could lead to depressive feelings [13,14]. Studies have consistently found that frequent distractions from smartphones are associated with negative emotional states, including depression and anxiety [15,16]. A study conducted in China highlighted that SD was significantly associated with problematic social media use, anxiety, depression, and stress [17]. Specifically, problematic social media use mediated the relationship between SD and anxiety as well as depression. Furthermore, cross-cultural studies involving Chinese and Korean college students demonstrated that the frequency of smartphone use positively predicted the severity of depressive symptoms [18,19]. Therefore, we are committed to exploring the underlying mechanisms of the link between SD and depression, which can lead to more effective interventions and support systems tailored to the unique needs of college students.

### 1.2 The mediating role of social withdrawal

Social withdrawal is defined as long-term (at least 6 months) social isolation and self-exclusion, including avoidance or absence of social activities, and decreased social interactions [20]. Notably, social withdrawal has become increasingly prevalent among college students [[21], [22], [23]]. Studies suggest that excessive smartphone use may reduce involvement in real-world activities and academic commitments, potentially leading to social withdrawal [24]. Individuals with limited offline interactions often seek social fulfillment through social media, which may foster smartphone dependence and exacerbate negative emotions [25]. While seemingly a solution, this shift can paradoxically deepen their reliance on smartphones, creating a vicious cycle where digital interactions replace genuine social engagement. Moreover, avoiding real-life social interactions and excessive reliance on digital devices can weaken the social support networks essential for managing stress among college students [26]. Dysregulated stress may increase the susceptibility to depression [27]. Given this context, this study seeks to investigate whether social withdrawal mediates the relationship between SD and depression in college students. Understanding this mediation can inform targeted intervention strategies to reduce social withdrawal and enhance real-world social interactions.

### 1.3 The mediating role of digital stress

Digital stress refers to the anxiety and stress caused by the notification and use of information and communication technologies of mobile phones and social media [28]. While digital media offer educational and personal development opportunities, they also pose risks to mental health [29]. From the JD-R model perspective, excessive notifications and continuous interaction with digital media represent job demands that exhaust an individual's limited cognitive and emotional resources [30]. When the volume of information to be processed surpasses an individual's capacity, it leads to digital stress. This overload can disrupt efficient information management and trigger both psychological and physiological stress responses, which may escalate into depressive symptoms. Empirical research supports this theory, for instance, Nick et al. [31] found that digital stress could longitudinally predict the development of depressive symptoms. Elisha et al. [32] demonstrated that frequent smartphone use may exacerbate depression by intensifying digital stress. Additionally, Wu et al. [33] observed that intensive social media use was linked with negative social comparisons, and increased depressive symptoms in college students. Based on this evidence, we hypothesized that digital stress as a mediating variable may affect the relationship between SD and depression in college students. By understanding this mediation, targeted intervention strategies can be developed to reduce digital stress and its negative impact.

### 1.4 The chain mediating effect of social withdrawal and digital stress

The two sources of mediators proposed above are also closely associated with each other. It has been shown that individuals at risk of Hikikomori often turn to virtual environments to escape reality [34]. Such habitual behavior, however, may gradually reduce their capability of real-life social interaction, leading to an increased reliance on online communication [35]. This shift initiates a vicious cycle where the more one withdraws socially, the more they may be exposed to digital stress [13,14]. Specifically, individuals who are frequently distracted by smartphones tend to experience more social withdrawal, which can then induce digital stress and consequently increase the risk of depression. This is consistent with the Stress Generation Models [36]. Therefore, we hypothesized that social withdrawal and digital stress serve as mediators for mediating the relationship between SD and depression.

### 1.5 The current study

Although previous research has documented the adverse effects of smartphone use on college students, significant gaps remain in our understanding, particularly concerning SD and its impacts on mental health, such as depression [37]. In fact, SD has become frequent and endemic among college students. Evidence underscores that distraction can lead to negative outcomes, including depression and anxiety [15,16], and poor sleep quality [38]. This underscores the need for a comprehensive investigation into the relationship between SD and depression. Moreover, existing studies suggested that SD can cause social withdrawal, digital stress, and depression; however, the relationship between the three variables caused by SD has not been clarified.

**Fig. 1:**
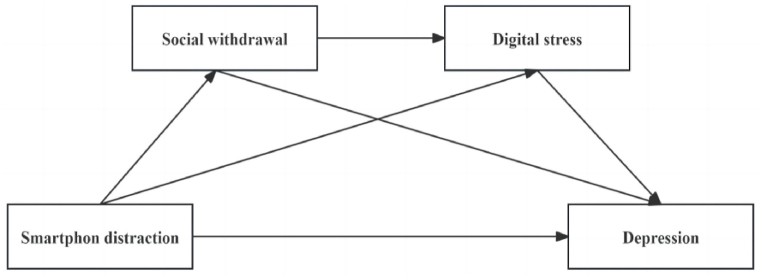
The proposed serial mediation model.

To address these gaps, we have developed a theoretical hypothesis model (Fig. 1), which will be tested to ascertain: (1) SD is significantly and positively correlated with depression of college students; (2) Social withdrawal plays an independent intermediary role between SD and depression of college students; (3) Digital stress plays an independent intermediary role between SD and depression of college students; (4) Social withdrawal and digital stress play a chain intermediary role between SD and depression. In conclusion, this study aims to further explore the relationship between SD, social withdrawal, digital stress, and depression in college students. This study will analyze the relevant factors and mechanisms affecting depression in college students, and further reveal the chain mediating effect of social withdrawal and digital stress between SD and depression, to provide the theoretical basis for preventing and reducing depression in college students.

## 2 Methods

### 2.1 Participants

The survey was conducted in seven colleges of Wuhan, Hubei Province, from September to November 2021. We utilized the wenjuanxing platform (https://www.wjx), a popular online tool for survey creation and data analysis in China, to facilitate the collection of responses. To incentivize participation and ensure data quality, the platform randomly selected 50 % of the participants to receive a small cash reward. The survey link or QR code was disseminated through WeChat, an extensively used app for messaging and social media in China. And QR code questionnaires were distributed through some of the relevant university teachers.

The research content was explained and informed digital consent was signed before any data collection. Each participant had the right to withdraw the survey at any time. 1319 participants completed the questionnaire. To ensure the quality of the data, an attention check question was incorporated into the survey. This question was designed to identify participants who were not fully engaged. Participants who failed the attention check were automatically flagged by the survey system, and their responses were excluded from the final analysis. Additionally, datasets from participants who declined to complete the questionnaires or whose completion time was too short (less than 5 min) were also excluded. Finally, 1184 participants (692 males and 492 females) were included. The mean age was 20.30 years (SD = 1.94; range = 17–37). Of these, 445 (37.58 %) were majoring in medical fields and 739 (62.42 %) in non-medical fields. Before the study, informed consent was presented to each participant, and obtained their informed consent. The effective response rate was 89.76 %. We ensured the confidentiality of the data and personal information of all participants. The study was approved by the Research Ethics Committee of Hubei University of Traditional Chinese Medicine (2021-ICE-015).

### 2.2 Sample size

A power analysis was conducted to determine the minimum sample size required for this study using a sampling formula cited by Bipolarity [39]. The significance level was set at α = 0.05, balancing accuracy with the practicality of gathering a sufficiently large sample. The z-value was set at 1.96, aligning with a 95 % confidence level. Based on a meta-analysis, the prevalence (*p*) of depression was estimated at 28.40 % [1]. A margin of error (d) was set at 5 % (0.05), ensuring that the study findings are robust and reliable without necessitating an unwieldy sample size.

$$n=\frac{\left(Z_{\frac{\alpha }{2}}\right)p\left(1‐p\right)}{d^{2}}$$

Considering a typical 20 % sample attrition rate observed in similar cross-sectional studies with college populations [40], we calculated that at least 376 questionnaires needed to be distributed.

### 2.3 Measurements

#### 2.3.1 Smartphone Distraction Scale (SDS)

SD was assessed by the SDS [37]. It comprises 16 items covering four dimensions: attention impulsivity, online vigilance, multitasking, and emotional regulation, which are scored on a five-point scale, ranging from 1 (rarely seldom) to 5 (almost always), with 3 indicating “sometimes”. The overall score ranges from 16 to 80, with higher scores indicating a greater inclination toward SD. The Chinese version of the SDS demonstrates good reliability [41]. The Cronbach's alpha for the SDS in this study was 0.920.

#### 2.3.2 25-Item Hikikomori Questionnaire (HQ-25)

Social withdrawal was assessed by the HQ-25 [42], which was originally developed to measure social withdrawal, specifically targeting the phenomenon of Hikikomori in adolescents. It comprises 25 items covering three dimensions: socialization, isolation, and emotional support, which collectively capture the essence of social withdrawal. Respondents rate each item on a five-point scale from 0 (strongly disagree) to 4 (strongly agree), with seven items requiring reverse scoring. The total score is obtained by summing all items, resulting in a potential score range of 0–100. The HQ-25 has been utilized and validated by Chinese college students [43]. In this study, the Cronbach's alpha for the HQ-25 was 0.884, reflecting its reliability.

#### 2.3.3 Multidimensional Digital Stress Scale (DSS)

Digital stress was assessed by the DSS [44]. It comprises 24 items covering five dimensions: availability stress, recognition anxiety, fear of missing out, connection overload, and online vigilance. Each item is scored on a 5-point scale, ranging from 1 (never) to 5 (always). The DSS score is the aggregate of all items. Higher scores indicate higher digital stress. Xie et al. [45] found that Cronbach's alpha for the scale was 0.937, and in our study, the reliability was slightly higher at 0.959.

#### 2.3.4 Patient Health Questionnaire-9 (PHQ-9)

Depression was assessed by the PHQ-9 [46,47]. There are 9 items in total. The questionnaire encompasses several key domains of depressive symptoms: emotional symptoms (e.g., "Feeling down, depressed, or hopeless"), cognitive symptoms (e.g., "Trouble concentrating on things"), physical symptoms (e.g., "Feeling tired or having little energy"), and thoughts of self-harm (e.g., "Thoughts that you would be better off dead, or of hurting yourself"). Each item has a score of 0 (not at all)-3 (almost every day), with a total score of 0–27. The higher score indicates the more serious depression symptoms. Among them, 0–4 points indicate no obvious depressive symptoms, 5–9 points are for mild depression, 10–14 points indicate moderate depression, 15–19 points are for severe depression, and ≥20 points denote extremely severe depression. In this study, the Cronbach's alpha for the PHQ-9 was 0.942.

### 2.4 Statistical analysis

SPSS 27.0 was used for descriptive statistics. Pearson's correlation coefficient was used to describe the correlation between measured variables. To evaluate common methodological biases, Harman's one-factor test was conducted, confirming that multiple factors influenced the data, which mitigates concerns regarding common method variance [48]. This enhances the robustness and validity of our conclusions. Additionally, the Variance Inflation Factor (VIF) was used to measure multicollinearity [49]. Mediation analysis was performed using Mplus 8.7, which facilitated robust testing of indirect effects via bootstrapping with 5000 samples. This method was selected to improve the accuracy of the confidence intervals (CI) for mediation effects, which are deemed significant if the 95 % CI does not include zero [50]. Additionally, the model controlled for age, gender, grade, major, and birthplace [[51], [52], [53]], as these variables are known to significantly influence mental health outcomes. In the context of China, it is also crucial to include educational level as a control variable, as graduate students often face more substantial academic and life pressures compared to their undergraduate counterparts. These pressures, which are often due to intense academic demands, high research expectations, and the stress of future employment prospects, are associated with elevated depressive symptoms [54]. In summary, age, gender, qualification, grade, major, and birthplace were included as control variables in the model.

## 3 Results

### 3.1 Participants characteristics

In our study, the mean age was 20.30 years (SD = 1.94; range = 17–37). Of all the included participants, there were 492 (41.55 %) males and 692 (58.45 %) females. Freshmen constituted 29.90 % of the sample, sophomores 24.49 %, juniors 32.18 %, seniors 8.78 %, fifth-year students 1.18 %, and postgraduates 3.46 %. Additionally, we found that 77.20 % (n = 914) of the participants reported depressive symptoms as measured by the PHQ-9. It is crucial to note, however, that this percentage does not necessarily indicate clinically significant levels of depression. The mean PHQ-9 score was 10.29 ± 6.33. More details can be found in Table 1.

**Table 1:** Characteristics of the study sample (n = 1184).

Variable	Category	N	Mean ± SD /Percentage(%)
Age			20.30 ± 1.94
Gender	Male	492	41.55
	Female	692	58.45
Grade	Freshman	354	29.90
	Sophomore	290	24.49
	Junior	381	32.18
	Senior	104	8.78
	Fifth-year	14	1.18
	Postgraduates	41	3.46
Major	Medical	445	37.58
	Non-medical	739	62.42
Birthplace	Urban	590	49.83
	Rural	594	50.17
Being the Only-Child	Yes	485	40.96
	No	699	59.04
Daily Smartphone Usage Hours	<3h	51	4.31
	≥3h – <6h	355	29.98
	≥6h – <9h	447	37.75
	≥9h	331	27.96
Depression Symptoms	Yes	914	77.20
	No	270	22.80

### 3.2 Common method bias testing

In this study, we utilized Harman's one-factor test to evaluate the potential for common method bias. This examines whether a substantial amount of variance can be attributed to a single factor. We extracted a total of 10 factors with eigenvalues >1. The first factor explained 36.61 % of the variance, falling below the often criterion of 40.00 %. While this result suggests that common method bias is unlikely to significantly distort our findings, several caveats remain. The 40.00 % cutoff is not definitive, and the test may overlook subtle biases distributed across multiple factors. Moreover, the test assumes unidimensionality, an assumption that may not hold in complex models. Therefore, despite the low indicated risk of serious common method bias, we should view these results with caution and employ additional analytical methods to comprehensively assess potential biases in the study.

### 3.3 Testing for multi-collinearity

The covariance diagnostics showed that the VIFs for SD, social withdrawal, and digital stress were 1.42, 1.46, and 1.12, respectively, which are all below the threshold of 5 [49]. Thus, multi-collinearity may not affect our estimates.

### 3.4 Descriptive analysis and correlations between overall variables

**Fig. 2:**
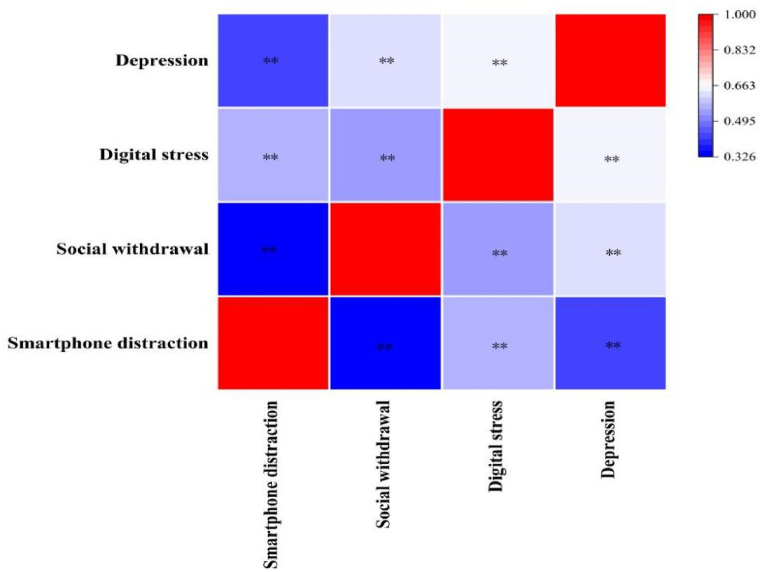
Correlation Heatmap of SD, social withdrawal, digital stress, and depression among college students. ^⁎⁎^*p* < 0.01.

The basic descriptive data for SD, social withdrawal, digital stress, and depression are shown in Table 2 and Fig. 2. The scores were as follows: SD was 49.59 (SD = 9.32), social withdrawal was 44.82 (SD = 13.30), digital stress was 69.71 (SD = 17.13), and depression was 10.29 (SD = 6.33). The results showed that SD was significantly positively correlated with social withdrawal (r = 0.327, *p* < 0.01), digital stress (r = 0.559, *p* < 0.01), and depression (r = 0.437, *p* < 0.01). In addition, social withdrawal was significantly positively correlated with digital stress (r = 0.544, *p* < 0.01) and depression (r = 0.625, *p* < 0.01). Lastly, digital stress was significantly positively correlated with depression (r = 0.646, *p* < 0.01).

**Table 2:** Correlations between the study variables (n = 1184).

	M	SD	1	2	3	4
1 Smartphone distraction	49.54	9.32	1			
2 Social withdrawal	44.82	13.30	0.327^a^	1		
3 Digital stress	69.71	17.13	0.559^a^	0.544^a^	1	
4 Depression	10.29	6.33	0.437^a^	0.625^a^	0.646^a^	1

Note: M, mean; SD, standard deviation.

a *p* < 0.01.

### 3.5 Mediation analysis of social withdrawal and digital stress

**Fig. 3:**
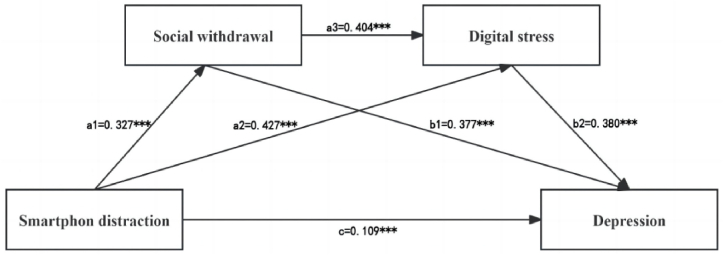
The chain mediation model of social withdrawal and digital stress in the relationship between SD and depression. Effects were reported as standardized values. ***p < 0.001.

To control for the potential confounding effects of demographic variables, such as age, gender, grade, major, and birthplace, we employed a uniform control approach by introducing all variables as covariates into the mediation model. However, the results indicated that the path coefficients for age were not statistically significant (*p* > 0.05). Thus, the non-significant paths were removed, and then the mediation model was re-estimated. The mediating effects of social withdrawal and digital stress are shown in Fig. 3. The fit indices for the modified model were acceptable [55]: χ^2^ = 44.487, d = 8. The Comparative Fit Index (CFI) was 0.980, well above the 0.950 benchmark for excellent fit. Similarly, the Tucker-Lewis Index (TLI) reached 0.954, confirming a good fit by accounting for model complexity. The Standardized Root Mean Square Residual (SRMR) stood at 0.051, significantly below the 0.080 threshold, and the Root Mean Square Error of Approximation (RMSEA) was 0.062, marginally exceeding the ideal value of 0.060 but remaining within the acceptable range of 0.050–0.080. These indices collectively affirm the model's robustness. The results showed that SD had a significant positive effect on depression (β = 0.109, *p* < 0.001), social withdrawal (β = 0.327, *p* < 0.001), and digital stress (β = 0.427, *p* < 0.001). Social withdrawal and digital stress had a positive effect on depression (β = 0.377, *p* < 0.001; β = 0.380, *p* < 0.001), respectively. The social withdrawal had a significant positive effect on digital stress (β = 0.404, *p* < 0.001).

The bootstrap 95 % CI indicates the significant indirect effects of social withdrawal and digital stress in the relationship between SD and depression (Table 3). These results suggested that social withdrawal and digital stress not only independently mediated the relationship between SD and depression but also had a chain-mediating effect on them.

**Table 3:** Bootstrap analysis of mediation effects (n = 1184).

Effect	Effect size	Percentage of total effects	BootLLCI	BootULCI
Total effect	0.299	100.00 %		
Direct effect	0.073^a^	24.41 %	0.037	0.108
Total indirect effect	0.226^a^	75.59 %	0.197	0.257
Indirect effect (X → M1 → Y)	0.083^a^	27.76 %	0.066	0.101
Indirect effect (X → M2 → Y)	0.109^a^	36.45 %	0.088	0.132
Indirect effect (X → M1 → M2 → Y)	0.034^a^	11.37 %	0.026	0.043

Note: Based on 5000 bootstrap resamples; Total, direct, and indirect effects of smartphone distraction (X) on depression (Y) through social withdrawal (M1) and digital stress (M2); CI: confidence interval.

a *p* < 0.001.

## 4 Discussion

This study explored the effects of SD on depression among Chinese college students, revealing a chain mediating effect through social withdrawal and digital stress. The findings revealed that SD not only disrupts immediate attention but also contributes to long-term mental health issues by exacerbating social withdrawal and digital stress, which in turn increase depression levels.

This study indicates a significant association between SD and depression among college students, aligning with previous studies [56,57] that affirm its negative impact on mental health. Utilizing the JD-R model as a theoretical foundation suggests that depression can emerge from an imbalance between personal demands and available resources [10]. In other words, SD drains cognitive resources essential for handling academic and personal demands [11], thereby increasing susceptibility to depression and reducing students' ability to engage effectively with their surroundings. Moreover, SD exacerbates depression by eroding social support networks [58]. While previous research showed the importance of strong social connections for stress management [59]; however, SD diminishes these networks and increases the risk of depression. Notably, Kaya et al. (2021) found a bidirectional relationship between depression and problematic smartphone usage such as smartphone addiction [60]. Although prolonged smartphone addiction may lead to SD, they are distinct social phenomena. Thus, a direct bidirectional relationship between SD and depression cannot be definitively inferred. Future studies can be explored in more depth. In conclusion, to mitigate these negative impacts, educational institutions should identify college students with excessive smartphone use early and provide timely interventions to minimize negative outcomes.

This study demonstrates that social withdrawal and digital stress independently mediate the relationship between SD and depression. Supporting the findings of Lee et al. [61] and Nick et al. [31], our research identifies social withdrawal and digital stress as significant risk factors for depression. Studies have found that SD leads to diminished attentional resources, impairing students' ability to handle cognitive demands efficiently and elevating their risk for depression. Specifically, SD undermines social interaction skills, hindering offline engagement and increasing the likelihood of social withdrawal among college students [62,63]. For college students, such negative lifestyle changes diminish social connections, thereby increasing the risk of depression. However, the results differ from those who suggested that social avoidance didn' t link to depression directly [64]. Indeed, social avoidance and social withdrawal are associated but different concepts. These inconsistencies may be due to differences in study variables and measurement tools. Additionally, prolonged SD can reduce the ability to conduct activities effectively, leading to digital stress [31]. This stress, arising from the constant demand to process digital information, significantly contributes to depression [65]. Therefore, educational institutions should not only foster diverse social activities to strengthen students' social connections but also guide managing digital engagement effectively to alleviate cognitive overload and minimize digital stress.

The key finding of our study is that social withdrawal and digital stress play a chain intermediary role in the relationship between SD and depression, which was not well explored in previous studies [66]. This chain mediation can be explained by the Parasympathetic Compensation Hypothesis [67], which proposes that individuals compensate for inadequate offline interactions by engaging excessively in online activities to satisfy their unmet social needs. This compensatory behavior typically increases smartphone usage among college students, resulting in greater distractions and avoidant behaviors. As a consequence, students may rely excessively on digital interactions, escalating their experience of digital stress. According to the Theory of Vicarious Internet Use [68], this dependency increases an overload of digital information, which intensifies digital stress and consequently, the risk of depression. The sequential nature of this model suggests that social withdrawal leads to increased digital stress, establishing a cyclical vulnerability where each component intensifies the next, ultimately heightening the risk of depression. This insight highlights the urgent need for interventions targeting both the behavioral patterns of smartphone use and the cognitive impacts of digital engagement.

Despite the insights provided by this study, some areas warrant further exploration in future research. First, it utilized convenience sampling, primarily recruiting from medical universities, which may compromise the diversity and representativeness of our sample. The predominance of female participants may lead to gender bias, potentially affecting the generalizability of the findings across different populations. Future studies should strive for a more balanced gender distribution and a wider range of educational backgrounds. Second, the cross-sectional nature of the study limits our ability to infer causality among the variables. Future research would benefit from longitudinal designs to more accurately validate the mediating roles of social withdrawal and digital stress. Finally, this study affirms the established relationships among SD, social withdrawal, and digital stress. Nonetheless, recent studies [14,69], have begun to suggest the possibility of bidirectional relationships between social withdrawal and digital stress. Future studies should investigate the relationship mechanism.

## 5 Conclusions

This study illuminates the pathways of how SD influenced depression in college students, highlighting the mediating roles of social withdrawal and digital stress. While the direct impact of SD on depression is relatively modest, the indirect effects mediated by social withdrawal and digital stress significantly magnify this relationship. The result suggests a mediating relationship where increased SD positively correlates with heightened social withdrawal and digital stress, further exacerbating depressive symptoms. The correlation between SD and depression suggests that more targeted interventions should be undertaken to relieve negative emotions, especially the depression of college students. However, given the limitations posed by the sample size in this study, further research involving larger sample sizes and longitudinal designs is crucial to understand and validate these relationships more comprehensively.

## Data availability

The datasets generated and/or analysed during the current study are not publicly available due privacy but are available from the corresponding author on reasonable request.

## Ethical approval statement

The study was approved by the Research Ethics Committee of Hubei University of Chinese Medicine (2021-ICE-015).

## Funding

This study was supported by 10.13039/501100003819Hubei Provincial Natural Science Foundation and Traditional Chinese Medicine Innovation and Development Joint Fund [Grant Number: 2023AFD160].

## CRediT authorship contribution statement

**Yufei Qiu:** Writing – original draft, Methodology, Formal analysis, Conceptualization. **Xueyang Zhao:** Writing – original draft, Methodology, Investigation, Formal analysis. **Jiali Liu:** Supervision, Project administration, Methodology. **Zhaoyang Li:** Project administration, Investigation. **Man Wu:** Investigation, Formal analysis. **Lixin Qiu:** Writing – review & editing, Supervision, Conceptualization. **Zhenfang Xiong:** Methodology, Funding acquisition, Formal analysis, Data curation. **Xiaopan Wang:** Supervision, Resources, Methodology, Funding acquisition, Formal analysis, Data curation. **Fen Yang:** Writing – review & editing, Supervision, Funding acquisition, Data curation, Conceptualization.

## Declaration of competing interest

The authors declare that they have no known competing financial interests or personal relationships that could have appeared to influence the work reported in this paper.
